# Editorial: Novel Therapeutic Targets and Emerging Treatments for Fibrosis

**DOI:** 10.3389/fphar.2017.00824

**Published:** 2017-11-14

**Authors:** Chrishan S. Samuel, Timothy D. Hewitson

**Affiliations:** ^1^Cardiovascular Disease Program, Biomedicine Discovery Institute and Department of Pharmacology, Monash University, Clayton, VIC, Australia; ^2^Department of Nephrology, Royal Melbourne Hospital, Parkville, VIC, Australia; ^3^Department of Medicine, Royal Melbourne Hospital, University of Melbourne, Carlton, VIC, Australia

**Keywords:** fibrosis, fibrogenesis, collagen, pharmacology, therapeutics

During development and wound healing, physiological fibrogenesis maintains connective tissue integrity and structure through synthesis of extracellular matrix (ECM). However, although many organs have a capacity to repair and regenerate after acute or mild injury, severe or ongoing stress results in fibrosis, which refers to the overgrowth, hardening, and scarring of tissues and ultimately causes a progressive loss of organ function (Wynn, 2007). Fibrosis results from an excess accumulation of scleroproteins, mainly collagen, which replaces parenchyma and stiffens tissues. This is in turn a consequence of both pathological fibrogenesis and the physical properties of scleroproteins, namely their aqueous insolubility, tendency to aggregate, and resistance to proteolytic digestion. Unfortunately, current therapeutic strategies for fibrosis are often indirect and non-specific, and only slow progression by a matter of months.

Whilst the pathology of fibrosis and its functional significance are well-understood, until recently we have known little about its molecular regulation. These mechanisms and their subsequent therapeutic targeting is the subject of this Research Topic. This themed issue therefore comprises several up-to-date mini-reviews and original studies on currently known and emerging therapeutic targets for preventing fibrosis and enhancing repair. In each case, mechanistic insights into how each of these factors contributes to regulating fibrosis progression is described, along with how they can be targeted by existing drugs, new mimetics, and cell-based therapies.

## Progression

While fibrosis represents a final common pathway to chronic and severe injury, we also know that the trajectory to organ failure can be highly variable (Li et al., 2012). The first article in this Research Topic therefore discusses the role of intrinsic and extrinsic risk factors in determining both susceptibility to injury and rate of progression, using chronic kidney disease as an example (Hewitson et al.). The role of epigenetic mechanisms in this process is emphasized here and in a study of histone marks in experimental kidney fibrosis (Hewitson et al.).

## Hypoxia and ischemia

Local tissue hypoxia is a feature of fibrotic disorders in many organs although it is often unclear whether hypoxia is a consequence or cause of fibrosis (Darby and Hewitson, 2016). Destruction of capillaries through accumulation of ECM results in a loss of oxygen delivery, local tissue hypoxia and upregulated hypoxia-inducible factor (HIF)-1α signaling. These aberrant processes in turn further upregulate ECM production in a vicious cycle. Xiong and Liu address the relationship between vascularization and fibrosis in disease, focusing on targeting HIF-1α. Related to this Nistri et al. explore the significance of Notch1 signaling in cardiac ischemia.

## Ligand-receptor relationships and molecular mechanisms

Central to our understanding of fibrosis has been the recognition that transforming growth factor beta (TGF-β1) is key to both the fibrogenesis and the perpetual activation of collagen-producing cells that occur in chronic conditions (Meng et al., 2016; Yue et al., 2017). Much of the focus in this Research Topic is therefore on the role of TGF-β1 signal transduction in fibrogenesis. Accordingly, Walton et al. discuss the contribution of TGF-β1 ligands to the pathogenesis of fibrosis. The significance of local TGF-β1 storage (Walton et al.) and its autocrine actions (Hewitson et al.) are likewise discussed. Caveolin has emerged as a crucial regulator of TGF-β1 signal transduction through TGF-β1 receptor internalization and degradation. This specific role and the more general significance of Calveolin-1 is reviewed in detail by Shihata et al.

For many years we have also realized that various members of the circulating and local renin-angiotensin-aldosterone system are directly and indirectly pro-fibrotic. Foremost are the fibrogenic actions of angiotensin II (Ang II) and aldosterone via binding to the angiotensin II type1 receptor (AT_1_R) and mineralocorticoid receptor (MR), respectively. Indeed, in many organs, Ang II and aldosterone inhibition and blockade are currently the best available anti-fibrotic therapies (Fang et al.). However, we now also realize that these ligand receptor families are more complex than first thought. While activation of AT_1_R by Ang II mediates vasconstriction, inflammation, and fibrogenesis, activation of the Ang II type 2 receptor (AT_2_R) has counter-regulatory vasodilatory, anti-inflammatory, and anti-proliferative effects. Wang et al. therefore examine the significance of the AT_2_R in fibrosis.

Cardiovascular and renal diseases are also associated with dysregulation of MR signaling, which has direct fibrotic effects, only some of which are related to aldosterone. Consistent with this Tesch and Young provide an overview of MR activation and signaling.

The bioactive lipid sphingosine 1-phosphate (S1p) intriguingly acts as both an intracellular signal transduction mediator, and when exported, a ligand for G protein-coupled receptors. In their mini-review Vestri et al. discuss the biology of S1p, its relevance to fibrogenesis, and effects of its modulation, including off target actions.

The pleiotropic properties of adenosine are also directly relevant to fibrosis. Again, however, ligand-receptor interactions are complex and context specific with pro-fibrotic and anti-fibrotic actions possible. Vecchio et al. explore the current understanding of the role of the most abundant adenosine receptor, A_2B_AR, in cardiac fibrosis.

## Therapeutic strategies

In many cases authors go on to discuss possible therapeutic approaches based on the above mechanisms. In their review of novel anti-fibrotic therapies, McVicker and Bennett provide several examples including microRNA, peroxisome proliferator-activated receptors, and strategies that directly target TGF-β1 signaling, including the endogenous inhibitors relaxin and bone morphogenic protein-7. The last of these is also discussed by Walton et al. Activation of the Notch1 pathway limits the extent of ischemic damage, promotes coronary angiogenesis and revascularization of the ischemic myocardium (Nistri et al.). In their mini review Wang et al. focus on pre-clinical testing of Compound 21, the most widely studied AT_2_R agonist in chronic disease, while Tesch and Young explore the therapeutic potential of MR receptor antagonism. Specific application of the S1p receptor agonist Fingolimod is examined by Ahmed et al.

However, despite the significance of fibrosis, restoration of organ function is more complex than simply removing or preventing fibrosis. Amelioration of fibrosis needs to be accompanied by tissue repair. Consistent with this stem cell therapies offer an exciting opportunity to enhance tissue repair in chronic organ disease (Lim et al.). The specific application of stem cell-derived exosomes is also demonstrated here experimentally in a rodent model of hepatic fibrosis (Alhomrani et al.).

Finally, this Research Topic provides a clinical perspective of various anti-fibrotic therapies for cardiovascular disease (CVD), outlining limitations of currently used therapies, the pipeline of anti-fibrotics for CVD, and why so many anti-fibrotic drugs have failed at the clinical level (Fang et al.).

## Conclusions

In conclusion, the recent identification of novel therapeutic targets, and the development of new treatment strategies based on them, offers the exciting prospect of more efficacious therapies to treat fibrosis. We sincerely thank the authors for their contributions and hope that each article in this Research Topic will both inform and generate further interest in this debilitating disorder.

## Author contributions

CS and TH wrote the Editorial and gave final approval for publication.

### Conflict of interest statement

The authors declare that the research was conducted in the absence of any commercial or financial relationships that could be construed as a potential conflict of interest.

## References

[B1] Darby I. A. Hewitson T. D. (2016). Hypoxia in tissue repair and fibrosis. Cell Tissue Res. 365, 553–562. 10.1007/s00441-016-2461-3 27423661

[B2] Li L. Astor B. C. Lewis J. Hu B. Appel L. J. Lipkowitz M. S.. (2012). Longitudinal progression trajectory of GFR among patients with CKD. Am. J. Kidney Dis. 59, 504–512. 10.1053/j.ajkd.2011.12.009 22284441 PMC3312980

[B3] Meng X. M. Nikolic-Paterson D. J. Lan H. Y. (2016). TGF-beta: the master regulator of fibrosis. Nat. Rev. Nephrol. 12, 325–338. 10.1038/nrneph.2016.48 27108839

[B4] Wynn T. A. (2007). Common and unique mechanisms regulate fibrosis in various fibroproliferative diseases. J. Clin. Invest. 117, 524–529. 10.1172/JCI31487 17332879 PMC1804380

[B5] Yue Y. Meng K. Pu Y. Zhang X. (2017). Transforming growth factor beta (TGF-beta) mediates cardiac fibrosis and induces diabetic cardiomyopathy. Diabetes Res. Clin. Pract. 133, 124–130. 10.1016/j.diabres.2017.08.018 28934669

